# Voltammetric analysis for fast and inexpensive diagnosis of urinary tract infection: a diagnostic study

**DOI:** 10.1186/s12967-018-1393-y

**Published:** 2018-01-25

**Authors:** Diana Lelli, Claudio Pedone, Pamela Alemanno, Alessandra Bertini, Claudia Di Gioia, Sara Fazzina, Giorgio Pennazza, Marco Santonico, Simone Grasso, Alessandro Zompanti, Silvia Angeletti, Raffaele Antonelli Incalzi

**Affiliations:** 1 Geriatric Unit, Campus Bio-Medico University of Rome, Via Alvaro del Portillo 200, 00128 Rome, Italy; 2 Unit of Electronics for Sensor Systems, Faculty of Engineering, Campus Bio-Medico University of Rome, 00128 Rome, Italy; 3 Medical Laboratory Unit, Campus Bio-Medico University Hospital, 00128 Rome, Italy

**Keywords:** Urinary tract infection, Voltammetric analysis, Dipstick test, Urine culture

## Abstract

**Background:**

Dipstick test is widely used to support the diagnosis of urinary tract infections (UTI). It is effective in ruling out UTI, but urine culture is needed for diagnosis confirmation. In this study we compared the accuracy of voltammetric analysis (VA) with that of DT to detect UTI (diagnosed using urine culture), and its usefulness as a second-stage test in people with positive DT.

**Methods:**

142 patients were enrolled with no exclusion criteria. VA was performed using the BIONOTE device. Partial Least Square Discrimination Analysis was used to predict UTI based on VA data; diagnostic performance was evaluated using sensitivity, specificity, positive and negative predictive values (PPV and NPV, respectively), positive and negative likelihood ratios (LR), accuracy, diagnostic odds ratio (DOR).

**Results:**

Mean age was 76.6 years (SD 12.6), 57% were male. VA had a better overall performance respect to DT in detecting UTI with accuracy 81.7% vs 75.9%, specificity 90.8% vs 82.5%, PPV 75% vs 61.4%, positive LR 6.68 vs 3.5, DOR 17.7 vs 7.47; sensibility, NPV and negative LR of the two tests were similar. VA had an accuracy of 82.4% in discriminating bacterial from fungal infections. When added as a second-stage test, VA identified 9 of the 17 false positive patients, with a net specificity of 91.7%, sensitivity 54%, PPV 75% and NPV 81%.

**Conclusions:**

VA is a quick and easy method that may be used as a second stage after DT to reduce the number of urine culture and of inappropriate antibiotic prescriptions.

## Background

Urinary tract infections (UTI) are the most frequent bacterial infections in the general population, with a worldwide incidence of about 120–250 million cases per year [1–3]. Not surprisingly, they also are the infectious disease that cause the highest global consumption of antibiotics [4], with inherent high risk of alterations of the normal vaginal and gastrointestinal microbiota, development of antibiotic resistance, and serious side effects such as such as *C. difficile* infections [5].

Compatible signs and symptoms are sufficient to make diagnosis of UTI in typical cases. However, the diagnosis should be supported by laboratory tests, such as physical and chemical dipstick test (DT) of urines. Urine culture represents the gold standard for the diagnosis of UTI, but it is often used only as a second-level diagnostic method because it is expensive and requires at least 48 h to deliver results. Urine culture and antibiogram are recommended in all males with suspected UTI and when there is suspected acute pyelonephritis, symptoms do not resolve or recur within 2–4 weeks after the completion of empirical treatment, and in women who present with atypical symptoms [6]. Currently, in case of urinary symptoms, the most common first line test for the diagnosis of UTI is the urine analysis, that can detect the presence of leukocyte esterase, nitrites or micro-haematuria. Data from a meta-analysis showed that this test has a pooled sensitivity of 81% (95% CI 71–90%), and a pooled specificity of 77% (95% CI 69–86%) [7], that is reduced by many condition, such as proteinuria, glycosuria, or bad preservation of the sample, but also varies according to the pathogen agent or patients’ dietary pattern (excessive vegetable intake); despite the aforementioned limitations, this test is effective in ruling out UTI, but its usefulness to rule in infection remains doubtful [6, 8, 9].

In the last 40 years, the interest towards sensory analysis techniques has considerably grown. Voltammetric analysis (VA) on liquid solution is one of such techniques that has already been applied to urine samples [10–16], both for detection of urinary creatinine and other compounds concentration [14, 15], and for detection of UTI [10, 12, 13]. These studies documented a good specificity in detection of UTI, but they used complex techniques that required several hours for sample preparation and analysis.

The objective of this study is to evaluate the accuracy of an innovative and portable e-tongue system for VA, able to directly work on urine samples and to provide results within a few minutes, to detect UTI compared to DT, using urine culture as the gold standard. Given that it is considered safe to rule out infections when a DT test is negative, this new methodology may have a role as a second-stage test in those having a positive DT, thus we also evaluated the role of VA in a two-stage testing approach. A secondary goal is to evaluate the ability of the e-tongue to identify fungal and bacterial infections.

## Methods

### Study design and population

This study was conducted at the Campus Bio-Medico Teaching Hospital in Rome, Italy, and was approved by the institution’s research ethics board (Protocol Number 22.16TS). We enrolled 142 consecutive patients admitted in the Geriatric and Hepatology Units between October 2015 and May 2016 that had performed an urine culture for clinical or laboratory signs of UTI. We had no exclusion criteria. Written informed consent was obtained from all participants.

### Specimens collection and analysis

Urine samples from the first voiding in the morning were analysed without any pre-treatment within 4 h after sampling in order to avoid refrigeration/re-warming cycles. The samples were collected in sterile cups.

Evaluation of leukocytes esterase and nitrite via urine DT was performed by Uropaper Alpha 3-9L (V-US29), Eiken Chemical CO., LTD; it was considered positive if leukocyte esterase or nitrites were present.

To perform semi-quantitative urine culture, 1 µl of freshly voided midstream urine was added to colistin-nalidixic acid blood agar and MacConkey agar, whereas 10 microliters were added to blood agar (Becton–Dickinson, Franklin Lakes, NJ, USA). Urine cultures with more than three species of bacteria or with < 10^4^ colony-forming unit/millilitre in patients with no symptoms (e.g. dysuria, urinary frequency, worsening incontinence, lower abdominal pain) or complications (e.g. haematuria, orchitis, urethritis, pyelonephritis, urosepsis) were considered not significant.

**Fig. 1:**
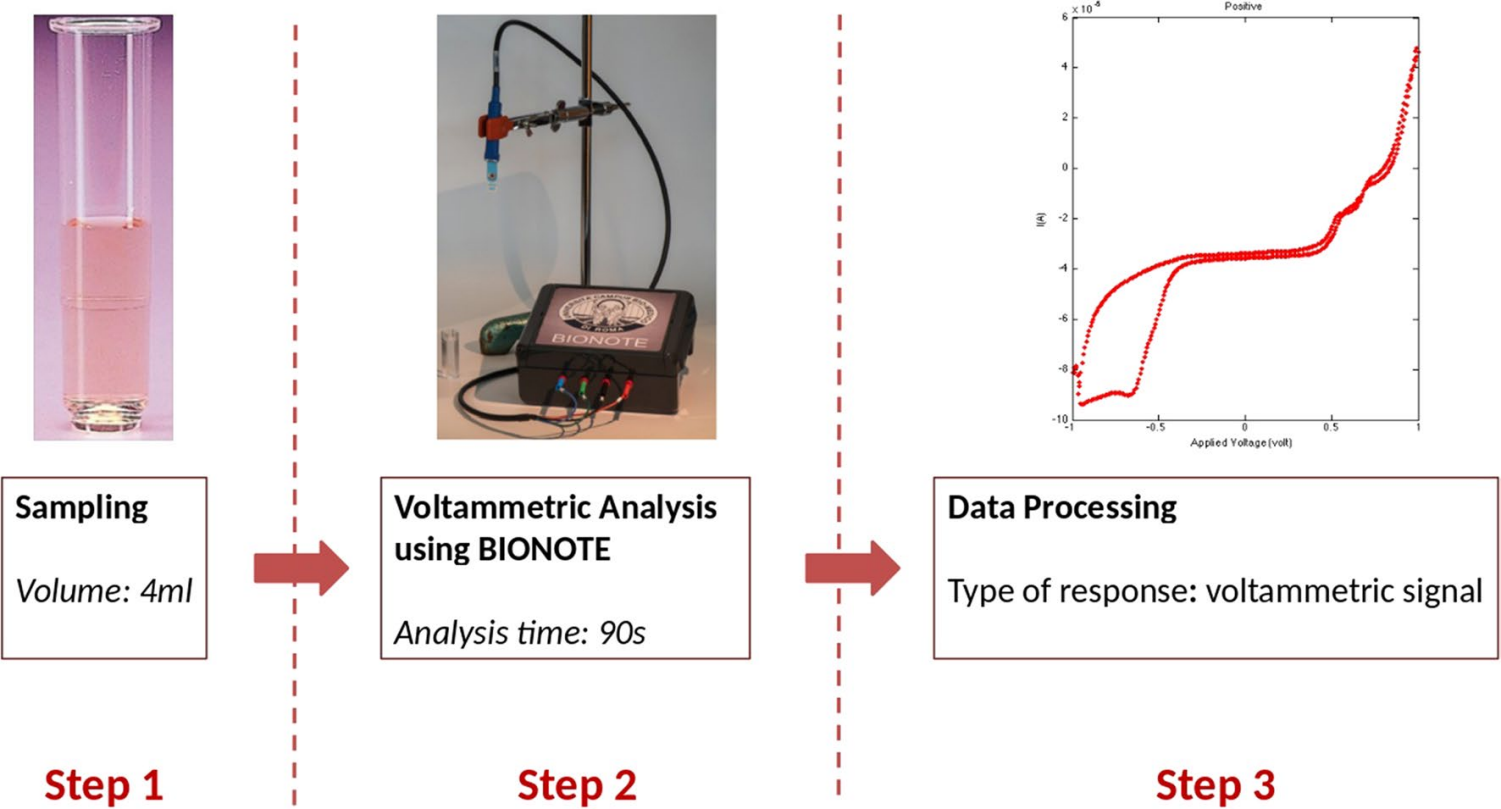
Experimental set up. A sample of 4 ml of urines is analysed using BIONOTE. This instrument provides a voltammetric signal, that differentiate infected from non-infected urines

VA was performed on the remainder of the urine sample by the innovative BIOsensor-based multisensorial system for mimicking NOse, Tongue and Eyes (BIONOTE). The liquid sensors of the BIONOTE consist of voltammetric screen printed electrodes controlled by a high stable electronic interface. Such stability is obtained by electronically settling the value of the reference electrode: this strategy can minimize the chemical drift of the reference electrode. Improving stability allows reliable response even to very small variations of the samples under measurement. The sensor probe consists of the typical configuration formed by three electrodes able to perform voltammetric analysis: working electrode, reference electrode and counter electrode (diameter of 4 ml) that are made of silver, platinum and gold, respectively. The working electrode takes the output signal as the sensor probe is immersed in a solution and the electrodes react changing the electrical configuration. The reference electrode offers a high impedance value and the current flows from the counter electrode to the working electrode, thus the impedance due to the reactions occurring in the sample influences this current. The applied input signal consists of a triangular waveform with a working range, from − 1 to + 1 V with 500 input voltages, the output is made up by the 500 corresponding output current values. The time duration of the complete measurement process was of about 90 s. All output data were stored in a flash memory. Data array was made up by the characteristic *fingerprints* extracted by each voltammogram registered for the measured samples. The set-up of the parameters for the acquisition, the number of samples and the sampling interval were controlled by a dedicated software interface [17] (Fig. 1).

### Analytic approach

The characteristics of the sample were reported using mean and standard deviation for continuous variables and proportion for categorical variables. The ability of BIONOTE to discriminate UTI was evaluated using Partial Least Square Discriminant Analysis with tenfold cross-validation [18]. The VA was also evaluated only in patients with positive DT, as this strategy is expected to reduce the false positive rate, that are a major concern in this context due to the risk of inappropriate antibiotic therapy. To evaluate the effectiveness of the diagnostic techniques we calculated sensitivity, specificity, positive and negative predictive values. We also calculated the positive and negative likelihood ratios (i.e. the ratio of true positives to false positives and of false negatives to true positives, respectively), that express how much the pre-test probability of the disease is affected by the result of the test. As overall measures of effectiveness we used the accuracy (i.e., the proportion of patients that were correctly classified), and the diagnostic odds ratio (DOR), that is the ratio of the odds of a positive test in diseased patients to the odds of a positive test in non-diseased patients. We used the same analytic approach to evaluate the ability of BIONOTE to discriminate bacterial vs. mycotic UTIs. All analyses were performed using R version 3.3.3 (R Foundation for Statistical Computing, Vienna, Austria).

## Results

Mean age of our study sample was 76.6 years (SD 12.6), 81 (57%) were male. The most prevalent diseases were arterial hypertension (91/144, 64%), diabetes (45/144, 32%), chronic renal failure (31/144, 22%) and liver disease (either cirrhosis or NASH, 18/144, 13%). DT was positive for esterase in 43 patients (30%), for nitrites in 10 patients (7%), and for esterase or nitrites in 44 patients (31%). Urine culture was positive in 44 participants, and in 8 (18.1%) the infection was sustained by two or more pathogens (Table 1). Fifty-two pathogens were identified (36 Gram-negative bacteria, 69.2%; 8 fungi, 15.4%; 8 Gram-positive bacteria, 15.4%). The most frequently isolated pathogen was the *Escherichia coli* (24, 46.2%), followed by *Candida albicans* (6, 11.5%), *Enterococcus faecalis* (8, 15.4%) and *Proteus mirabilis* (3, 5.8%) (Table 2). Other germs such as *Klebsiella pneumoniae*, *P. aeruginosa* spp., *Morganella morganii*, *Enterobacter cloacae*, *Cricobacter koseri*, *Acinetobacter baumannii* and other species of Candida, were isolated in a minority of patients (Morganella in 2 patients and 1 isolation for each other microorganism).

**Table 1:** General characteristics of the population

	Total sample (N = 142)
Age (years, mean [SD])	76.6 (12.6)
Male sex (N [%])	81 (57)
Comorbidities (N [%])	
Hypertension	91 (64)
Diabetes	45 (32)
Chronic kidney failure	31 (22)
Liver diseases	18 (13)
Dipstick test (N [%])	
Esterase	43 (30.5)
Nitrites	10 (7.1)
Positive urine culture (N [%])	44 (31)
Poly-microbial infection	8 (18.2)

**Table 2:** Microbiological characteristics of positive urine culture

	Identified pathogens (N = 52) N (%)
Gram+ infection	8 (15.4)
*Enterococcus faecalis*	8 (15.4)
Gram− infection	36 (69.2)
*Escherichia coli*	24 (46.2)
*Proteus mirabilis*	3 (5.8)
Other	9 (17.3)
Fungal infection	8 (15.4)
*Candida albicans*	6 (11.5)
Other	2 (3.8)

Table 3 shows the effectiveness of DT and VA in detecting UTI using urine culture as the gold standard. Overall accuracy of DT was 75.9% (95% CI 68–82.7%) with a DOR of 7.5 (95% CI 3.4–16.7); sensitivity was 61.4% (95% CI 45.5–75.6%), specificity 82.5% (95% CI 73.4–89.4%), PPV 61.4% (95% CI 45.5–75.6%) and NPV 82.5% (73.4–89.4%). The positive LR was 3.5 (95% CI 2.14–5.72) and the negative LR was 0.47 (95% CI 0.32–0.69). VA had a better overall performance, with accuracy 81.7% (95% CI 74.3–87.7%) and DOR 17.7 (95% CI 6.3–39.2). This was reflected by a better specificity (90.8%, 95% CI 83.3–95.7%) and PPV (75%, 95% CI 57.8–87.9%), while sensitivity and NPV were comparable to DT.

**Table 3:** Diagnostic performance of DT and e-tongue

	Dipstick	E-tongue
Accuracy, % (95% CI)	75.9 (68–82.7)	81.7 (74.3–87.7)
Sensitivity, % (95% CI)	61.4 (45.5–75.6)	61.4 (45.5–75.6)
Specificity, % (95% CI)	82.5 (73.4–89.4)	90.8 (83.3–95.7)
PPV, % (95% CI)	61.4 (45.5–75.6)	75 (57.8–87.9)
NPV, % (95% CI)	82.5 (73.4–89.4)	84 (75.6–90.4)
Positive likelihood ratio (95% CI)	3.5 (2.1–5.7)	6.7 (3.4–1.3)
Negative likelihood ratio (95% CI)	46.8 (31.9–68.7)	42.5 (29.2–62.1)
Diagnostic odds ratio (95% CI)	747.4 (335.4–1665.7)	1570.6 (628.7–3923.6)

*PPV* positive predictive value, *NPV* negative predictive value

With respect to the type of infection, VA was able to correctly identify 1 out of 8 patients with culture positive for fungi, 23 out of 36 patients with culture positive for bacteria, and 93 out of 98 of patients with negative culture, with an accuracy of 82.4% (95% CI 75.1–88.3%) (Table 3). Thus, this method had a sensitivity of 12.5% (95% CI 0–52.7%), a specificity of 100% (95% CI 97.3–100%), a PPV of 100% (95% CI 2–100%), and a NPV of 95% (95% CI 90–98%) in detecting fungal infection from sterile cultures, and a sensitivity of 63.9% (95% CI 46.2–79.2%), a specificity of 93.4% (95% CI 86.9–97.3%), a PPV of 77% (95% CI 57.7–90.1%), and a NPV of 88% (95% CI 81–93.7%) in detecting bacterial infection from sterile cultures. Of the 44 patients with positive DT, 17 (39%) were false positives. When VA was added as a second-stage test in participants with positive DT, 9 of the 17 false positive patients were correctly classified. The net specificity of the two-stage approach was 91.7% (95% CI 38.8–69.6%), with a loss of sensitivity, that was 54.5% (95% CI 38.9–69.6%), and the PPV and NPV were 81.7% (95% CI 73.1–88.4%) and 75% (95% CI 56.6–88.5%), respectively.

In the same group of patients, VA showed an accuracy of 93.2% (95% CI 81.3–98.6%) in correctly classifying the type of infection. This analysis correctly classified 2 of the 4 fungal infections and 22 of the 23 bacterial infections, showing a sensitivity of 95.7% (95% CI 78.1–99.9%), a specificity of 95.2% (95% CI 76.2–99.9%), a PPV of 95.7% (95% CI 78.1–99.9%) and a NPV of 95.2% (95% CI 76.2–99.9%) in discriminating bacterial infections from sterile urines and a sensitivity of 50% (95% CI 6.8–93.2%), a specificity of 100% (95% CI 91.2–100%), a PPV of 100% (95% CI 15.8–100%) and a NPV of 95.2 (95% CI 83.8–93.4%) in discriminating fungal infections from sterile urines (Table 4).

**Table 4:** Discrimination of type of infection

	All patients			
	Urine culture			
		Negative	Bacterial infection	Fungal infection
E-tongue	Negative	93	13	5
	Bacterial infection	5	23	2
	Fungal infection	0	0	1

	Patients with positive urinary dipstick			
	Urine culture			
		Negative	Bacterial infection	Fungal infection
E-tongue	Negative	17	1	1
	Bacterial infection	0	22	1
	Fungal infection	0	0	2

## Discussion

The analysis of electrochemical patterns in solution using BIONOTE seems to have better diagnostic performance compared to the DT, both overall and with respect to the PPV. Thus, this method can rule-out UTI as effectively as the DT, but is superior to the DT in correctly identifying patients with positive urine culture. As expected, a two-stage approach increases the net specificity compared to DT alone, although it is not superior to VA alone. Furthermore, compared to DT, VA has the additionally advantage of being able to provide information on the type of infection, that are significantly more accurate when used after a positive DT. In consideration of the large diffusion of UTI, the research of new techniques for quick and reliable diagnostic tests is very important, but it actually has not been highly productive. In this context, e-tongue is a technology with high potentialities, that was already tested on urine samples and documented interesting results in detecting UTIs. For example, Lamb et al. showed that an electrochemical method was able to detect 94% of the positive urine samples in 4 h, and 100% of them in 10 h, and all negative cultures [10]. Similarly, Cady et al. showed that an automated instrumental procedure based on changes in impedance related to bacterial growth, correctly classified 95.8% of urine cultures after 2.6 h, with 1.9% of false positive and 2.3% of false negative urine samples. A longer time of analysis reduced false negatives, but also an increase of false positives (4.9 and 1.2%, respectively) [12]. However, all of the previously described techniques required a relatively long examination time, and complex and bulky devices. Furthermore, none of these techniques was tested for identification of fungal infection.

In contrast, BIONOTE is a portable system, that allows a quick (about 10 min) and simple (it could be performed also by a non-specialist) analysis, and that allows to obtain repeatable and reproducible response for each potential applied; therefore this technology is innovative in the background. In this study, we demonstrated that VA is as effective as DT in excluding UTIs, but its cost (approximately 7€) is significantly higher than that of DT, therefore it is sensible to use it only as a second-stage test in those with positive DT, in order to reduce the costs related to false positives DT tests. Another innovative and important potentiality of BIONOTE is the ability to exclude fungal infections, especially when performed after a positive DT. Mycoses account for 1% of uncomplicated UTIs, that increase to 7% in complicated ones [19], and to 26% in catheterized patients presenting with nosocomial UTI [20]. Furthermore, urinary tract candidiasis is known as the most frequent nosocomial fungal infection worldwide [21]. Therefore, providing in few minutes accurate results, this technology would allow orientate the empirical therapy, and to exclude fungal infections, also in selected patients at greater risk of mycotic urinary infections such as immunodepressed or patients with indwelling catheter or exposed to frequent antibiotic therapies. In these patients, the incorrect use of an antibiotic instead of antifungal agent may increase the risk of fungal dissemination and sepsis. Thus, the timely recognition and correct treatment of these infections may substantially change prognosis of these patients.

A limitation of our study is that we analyzed a convenient sample of patients consecutively admitted to medical wards of a university hospital. However, the high accuracy of BIONOTE in discrimination of UTIs is related to the ability of the technology in detecting changes in chemical characteristics of the samples. Therefore, these preliminary results could also be applied to uncomplicated patients and to the general population. Another potential issue is that we compared VA with DT, as suggested by current guidelines [6] and not with urinary microscopy. As the latter method is more accurate than DT [22], our results may have been different if we had used it. Finally, there is a relatively small sample, with consequent few UTIs.

Despite these limitations, present findings seem to be highly representative of the UTI pattern. Indeed, confirming the existing literature, *E. coli* proved to be the most frequent etiological agent of the UTI in our series, followed by *C. albicans*, *E. faecalis* and *P. mirabilis*. Thus, the microbiological profile of our population is akin to that characterizing many samples representative of the general population [19].

## Conclusions

According to present data, the VA of the electrochemical pattern represents a quick and easy method, which may be used sequentially after the urinary DT to reduce the numbers of urine cultures. This technology allows improve the rule-in ability of the DT, thus reducing the risk of inappropriate antibiotic prescription and consequently the risk of development of antibiotic resistance. Furthermore, this technology has the added advantage of excluding a mycotic infection, that may help the choice of treatment until availability of urine culture results.

In the future, it would be useful to validate this methodology on a larger scale and possibly comparing it also with urinary microscopy, in order to provide the current results with larger statistical evidence. A further goal might be the development of an algorithm, which would allow the system be used as a diagnostic instrument.

## Abbreviations

UTI: urinary tract infection
DT: dipstick test
VA: voltammetric analysis
BIONOTE: BIOsensor-based multisensorial system for mimicking NOse, Tongue and Eyes
DOR: diagnostic odds ratio
